# Perceptions and practices for preventing malaria in pregnancy in a peri-urban setting in south-western Uganda

**DOI:** 10.1186/s12936-016-1246-1

**Published:** 2016-04-14

**Authors:** Anthony K. Mbonye, Said M. Mohamud, James Bagonza

**Affiliations:** Ministry of Health, Kampala and School of Public Health-College of Health Sciences, Makerere University, Box 7272, Kampala, Uganda; School of Public Health, College of Health Sciences, Makerere University, Kampala, Uganda

**Keywords:** Malaria in pregnancy, Sulphadoxine–pyrimethamine, Perceptions, Practices, Peri-urban, Uganda

## Abstract

**Background:**

Malaria in pregnancy contributes greatly to maternal morbidity and mortality in Uganda. Thus it is urgent to identify possible barriers that limit access to existing interventions. The aim of this study was to assess perceptions and practices regarding malaria prevention during pregnancy in a peri-urban area and explore ways to scale-up malaria prevention interventions, since little is known about malaria in peri-urban settings.

**Methods:**

A survey was conducted in Kabale municipality south-western Uganda from April–June, 2015. Data was collected using a structured questionnaire targeting pregnant women, who delivered in the study area 1 year prior to the survey. Univariate analyses were performed at assess the level of knowledge and practices on malaria prevention during pregnancy.

**Results:**

A total of 800 women was interviewed. The majority of women, 96.1 % knew that malaria was a dangerous disease in pregnancy; 60.3 % knew that it caused anaemia, and 71.3 % associated malaria with general weakness. However, fewer women (44.9 %) knew that malaria in pregnancy caused abortions, while 14.9 % thought it caused stillbirths. Similarly, few women (19 %) attended the recommend four antenatal care visits; less than a half (48.8 %) accessed two doses of sulfadoxine-pyrimethamine (SP) for malaria prevention in pregnancy while 16.3 % received at least three doses of SP, as recommended by the current policy. The main reasons for poor antenatal care attendance were: women felt healthy and did not see a need to go for antenatal care, long distances and long waiting hours at clinics. The reasons given for not taking SP for malaria prevention were: women were not feeling sick; they were not aware of the benefits of SP in pregnancy, they were sleeping under insecticide-treated nets; fear of side effects of SP; and the antenatal care clinics were far.

**Conclusion:**

Despite a good knowledge that malaria is a dangerous disease in pregnancy, there was poor access to antenatal care and use of SP for malaria prevention in pregnancy. There is urgent to address existing health system constraints in order to increases access to malaria prevention in pregnancy in this setting.

## Background

Uganda has a high burden of malaria in pregnancy that contributes greatly to maternal morbidity and mortality [1, 2]. The current policy is to give at least three doses of sulfadoxine–pyrimethamine (SP), as intermittent preventive treatment (IPTp) [3]. For treating malaria in pregnancy in first trimester, the policy is to give quinine 10 m/kg; and artemisinin-based combination therapy (ACT) during the second and third trimester [3].

Interventions to prevent malaria through the public sector have not achieved the desired targets. Recent data shows low utilization of essential health services resulting in missed opportunity. For example, 47.6 % pregnant women attend the required four antenatal care visits; 26.7 % receive the two doses of SP-IPTp while 55.5 % get at least two doses of tetanus toxoid vaccination [4]. The previous studies have assessed factors associated with low utilization of health-based interventions, including maternity services and have documented socio-cultural factors, economic, constraints, negative perceptions, poor quality of care and long distances to health units [5–11, 22, 23]. A recent survey in Uganda showed that financial constraints, long distances to the health facilities and concern that drugs were not available were the most significant factors affecting access to health care [4].

There are few epidemiological features of malaria in pregnancy relevant for research as well as deciding on control strategies. First, in malaria endemic areas, the frequency and severity of malaria is greater in pregnant women [12–16]. Second, the severity of clinical manifestations of malaria in pregnancy is determined by the level of pre-pregnancy immunity, which largely depends on the intensity and stability of malaria transmission. Thus, in areas where malaria transmission is low and unstable, the degree of acquired immunity of women is low and both the mother and the foetus may suffer severe consequences of the disease [12]. The third important feature is that in areas of high malaria transmission, primigravidae are more affected [14]. Because of the severe presentation of malaria in low endemic areas, it anticipated that pregnant women in such areas are aware of the severe manifestations and are likely to take preventive actions [17].

Studies on perceptions on malaria in Uganda have shown that malaria is perceived as a common disease [18, 19]. One study showed that the preventive actions recommended by the women were in line with their perceptions of cause. The use of herbs was mentioned as the first treatment action, followed by the purchase of tablets from shops, with the final recourse being the formal health sector if the previous actions had not led to cure [20]. The aim of this study, therefore, was to assess perceptions and practices on malaria prevention in pregnancy in a pre-urban area in Uganda and explore ways to scale-up these interventions.

## Methods

### Study design

A survey was conducted in Kabale municipality, south-west Uganda, from April to June, 2015. This area was selected because little is known about malaria in peri-urban areas. All the three divisions in Kabale municipality participated in the study. A division has a population of approximately 50,000 people and a ward 5000 people. Data collection targeted pregnant who delivered 1 year prior to the survey. The Local Council III offices provided data on divisions, wards and the number of households in each ward. In each ward, a list of households was obtained and all women aged 15–49 years, who had delivered 1 year preceding the survey and consented to participate in the study were interviewed.

### Study setting

The study was conducted in Kabale District, in south-western Uganda. The district experiences low malaria transmission and occasionally gets malaria outbreaks with severe signs and symptoms of the disease. It is situated 560 km from the capital city, Kampala and has temperatures ranging from 11 to 24 °C; with a mean annual rainfall of 1000–1250 mm. There are two rainy seasons, the heavy one between March and May, and the lesser one between September and November. Kabale municipality has a total of 32 health facilities including eight government health centres and one referral hospital. All the health facilities offer antenatal care (ANC), distribution of insecticide-treated nets (ITNs), malaria treatment and information on prevention of malaria.

### Data sources

Data was collected on the following variables: socio-demographic characteristics, access to ANC and delivery care, access to malaria prevention services, use of SP for IPTp, and knowledge on adverse effects of malaria and preventions practices. Five research scientists experienced in malaria research conducted the interviews. They underwent refresher training for 3 days on research techniques, and study procedures; and participated in the pretesting and revision of the questionnaire tools before actual field work.

The questionnaire was pretested among 43 participants outside the study area. Some questions on perceptions of malaria in pregnancy that had not been framed well and therefore not understood by the participants were revised for clarity. The final questionnaire was administered in the local language (Rukiga). Said Ishak supervised all aspects of data collection.

### Sample size calculation

Sample size calculation aimed to detect a difference of 5 % in the proportion of women who completed antenatal care visits in the district estimated at 38 % [21]. In order to estimate the proportion with a ±5 % absolute precision, at a power of 80 and 5 % level of significance (two-sided), a minimum sample of 784 pregnant women with 10 % non-participation was targeted for the study.

### Data analyses

Data was entered and verified using Microsoft Access 2007 (Microsoft Inc., Redmond, Washington) and analysed using STATA version 11.0 (STATA Corporation, College Station, Texas). Univariate analyses were performed to calculate proportions on perceptions and preventing practices. Responses from open-ended questions were coded and manually analysed.

### Ethics

Ethical approval for the research was granted by Makerere University, College of Health Sciences, School of Public health Higher Degrees, Research and Ethics Committee (HDREC). Permission was also granted by the District health office. During field work, an information sheet about the study was given out and written informed consent was sought prior to the interviews.

## Results

A total of 800 women who had delivered a year prior to the survey were interviewed. Their mean age was 29.8 years, range (16–49). The majority were married 701 (87.6 %) while 328 (60.5 %) had attained at least primary education. Women were involved in different activities, 207 (29.9 %) were trading, 281 (35.1 %) were peasant farmers while 155 (19.4 %) were housewives (Table 1).

**Table 1:** Socio-demographic characteristics of respondents

Socio-demographic characteristics	Frequency (%)
Mean age 29.8 range (16–49 yrs)	
Marital status	
Married	701 (87.6)
Divorced	32 (4.0 %)
Widowed	17 (2.1 %)
Separated	50 (6.3 %)
Educational level	
No education	54 (6.8 %)
Primary	297 (37.1 %)
Secondary	187 (23.4 %)
Tertiary	141 (17.6 %)
University	120 (15.0 %)
Occupation	
Housewife	155 (19.4 %)
Peasant farmer	281 (35.1 %)
Trading/business	207 (29.9 %)
Employed by the government	109 (13.6 %)

The majority of women, 769 (96.1 %) knew that malaria was a dangerous diseases in pregnancy, 469 (58.6 %) knew about HIV/AIDS, 405 (50.6 %) high blood pressure and 135 (16.9 %) knew about syphilis. Similarly, the majority of women, 482 (60.3 %) knew that anaemia was due to malaria in pregnancy, 359 (44.9 %) knew about abortions, 119 (14.9 %) knew about stillbirths and 570 (71.3 %) associated malaria in pregnancy with general weakness (Table 2).

**Table 2:** Knowledge and prevention practices on malaria in pregnancy

Knowledge and prevention practices	Frequency (%) N = 800
Knowledge on dangerous diseases in pregnancy	
Malaria	769 (96.1 %)
HIV/AIDS	469 (58.6 %)
Syphilis	132 (16.5 %)
High blood pressure	405 (50.6 %)
Diabetes	135 (16.9 %)
Knowledge on the negative effects of malaria in pregnancy	
Anaemia	482 (60.3 %)
Abortion	359 (44.9 %)
Still births	119 (14.9 %)
Poor health (general weakness)	570 (71.3 %)
Antenatal care attendance	
Attended at least once in the last pregnancy	538 (72.5 %)
Attended the recommended 4 visits	102 (19.0 %)
Attended ANC at public facility	451 (83.8 %)
Attended ANC at private facility	87 (16.2 %)
Postnatal care attendance	
Attended after six weeks of delivery	473 (59.1 %)
ITN Use	
Slept under and ITN a night before the interview	628 (78.5 %)
Taken SP for malaria prevention	
Taken SP	326 (40.8 %)
Taken the two recommended doses of SP	159 (48.8 %)
Taken three doses of SP	53 (16.3 %)
Taken four doses of SP	15 (4.6 %)

The majority of women, 451 (83.8 %) attended at least one ANC visit while few attended the four recommended visits, 102 (19.0 %). The majority received ANC from public facilities, while few, 87 (16.2 %) received ANC from private facilities (Table 2). ANC attendance was associated with the level of a woman’s education, *P* = 0.0001; the woman’s occupation status, *P* = 0.001; partners’ education level, *P* = 0.001; and the number of children a woman had, *P* = 0.002. Results further show that the majority of women, 628 (78.5 %) slept under an ITN a night before the survey; while less than a half, 159 (48.8 %) accessed two doses of SP. Few, 53 (16.3 %) received at least three doses of SP as recommended by the current policy (Table 2).

The study solicited reasons why women did not attend ANC and reasons for not taking SP for malaria prevention in pregnancy. The reason given by most women who did not attend ANC was that they were feeling well and they did not see any reason for attending ANC, 213 (81.3); other reasons were: ANC clinics were far, 193 (73.7 %), long waiting hours at clinics, 136 (51.9 %); and husbands did not approve, 42 (16.0 %) (Table 3).

**Table 3:** Reasons for not attending ANC and not taking SP as IPTp

	Frequency (%)
Reason for not attending ANC	N = 262
Was feeling healthy	213 (81.3 %)
Was busy	52 (19.9 %)
ANC clinic was too far	193 (73.7 %)
Long waiting hours at the clinic	136 (51.9 %)
Husband did not allow	42 (16.0 %)
There is poor quality at the clinic	27 (10.3 %)
Reasons for not taking SP for malaria prevention	N = 473
Because I was sleeping under an ITN	80 (16.9 %)
I was not sick	170 (35.9 %)
The health unit was far	123 (26.0 %)
There were no drugs at the ANC clinic	68 (14.4 %)
I was not aware of the use of SP	85 (18.0 %)
I did not have money	65 (13.7 %)
The health worker was busy	43 (9.1 %)
I feared side effects	38 (8.0 %)
I used herbs	25 (5.3 %)

The reason given by most women for not taking SP for malaria prevention in pregnancy was that they were not sick and, therefore, did not see any reason for taking SP, 170 (35.9 %); women were not aware of the benefits of SP in malaria prevention in pregnancy, 85 (18.0 %); and fear of side effects, 38 (8.0 %). Others were: the health units were far, 123 (26.0 %); because women were sleeping under ITNs, 80 (16.9 %); and drug stock-outs at health units, 68 (14.4 %) (Table 3).

## Discussion

The present results show that although there was high knowledge that malaria was a dangerous disease in pregnancy, few women knew that malaria in pregnancy caused abortions and stillbirths. Similarly, few women attended the recommend four ANC visits; while few received at least three doses of SP as IPTp as recommended by the current policy. The main reasons for poor ANC attendance were: women felt healthy and did not see a need to go for ANC, long distances to clinics and long waiting hours at clinics; while the reasons given for not taking SP for malaria prevention in pregnancy (IPTp) were: women were not feeling sick; they were not aware of the benefits of SP in pregnancy, fear of side effects of SP, they were sleeping under ITNs; and the ANC clinics were far. Thus there is urgent need to address these constraints in order to increases access to malaria prevention in pregnancy in the study area. Data in Uganda shows that there is relatively high ANC attendance (4 visits) and access to least two doses of tetanus toxoid; yet women who receive two does of SP-IPTp is low calling for interventions to reduce this missed opportunity. This scenario is consistent with findings of a study in sub-Saharan Africa that show missed opportunities through ANC [22]. Similarly, a previous study has documented constraints to access SP-IPTp such as high user fees, poor quality of care, frequent drug stock-outs and confusing polices and guidelines on SP-IPTp [23].

The present findings highlight the importance of how the awareness about a disease and the way people perceive themselves at risk of that disease influences the preventive actions taken as previously described in the Health Belief Model [17]. As shown, although women knew that malaria was a dangerous disease in pregnancy and caused anaemia, few associated it with loss of pregnancy through abortions and stillbirths. It is possible this perception contributed to few women taking SP for malaria prevention in pregnancy. To compound this, few women knew the benefits of SP and they feared side effects of SP.

The present results are consistent with findings of an earlier study in the same district which showed that ANC attendance was irregular and few women knew that the purpose of attending antenatal care was to monitor both the growth of the baby and the health status of the woman. Health-seeking behaviour was influenced by several factors, including the perceived high cost of ANC services, and perceived inadequacy of services provided by the formal health system. Inadequacy of formal health services was perceived by users to be partly due to understaffing and to irregular supply of essential drugs [20].

The present results are also consistent with findings of another study carried out in a high malaria endemic and largely a rural district in Uganda which showed that that primigravidae, adolescents and men were not considered at risk of malaria. Similarly anaemia and low birth weight were not associated with malaria; and most women could not differentiate symptoms of malaria from those of early pregnancy [19].

While interpreting these results there is need to be cautious about several limitations. This study was carried out in a mainly urban area and may not represent rural women who may have difficulties in accessing information and health services. Thus the perceptions and health-seeking practices could be different. Similarly, this was a rapid survey and did not explore fully perceptions and health-seeking behaviour related to malaria in pregnancy and this is recommended this for further studies.

The study in a peri-urban area is timely since little is known about malaria prevention practices in such settings. Yet with increasing urbanisation in Uganda, there is need to identify barriers that could help in designing delivery of malaria prevention interventions. It is shown that a high proportion of the study population had attained secondary education thus could appreciate health messages and the benefits of malaria prevention in pregnancy and possibly adopting positive behaviour practices. Since there has been wide distribution and use of ITNs in Uganda; (78.5 % in the study area); there is need for further studies to explore how the changing malaria endemicity will affect perceptions and practices in such settings.

The immediate policy implication of these results is create a health promotion package that explains the benefits of SP for malaria prevention in pregnancy, its dangers and the importance of attending ANC to receive treatment and prevention services; as previously recommended [24]. However, there was a substantial number (16 %) of women in the present study who were not allowed to attend ANC by their husbands. Thus engaging men in promoting maternal health should be part of this package.

These results will be discussed with the district health team to enable them design this package. The medium term policy implications is to discuss health system strengthening interventions to ensure improved quality of care and modalities of bring services close to pregnant women through regular outreach programmes.

In conclusion, these results show that there is still a disconnect between what women know about malaria, its negative effects and health-seeking behaviour. To increases access to malaria prevention in pregnancy, there is need to create both awareness on malaria in pregnancy as well strengthening the health system to provide quality and acceptable services in this district.
